# Chimerism Analysis of Cell-Free DNA in Patients Treated with Hematopoietic Stem Cell Transplantation May Predict Early Relapse in Patients with Hematologic Malignancies

**DOI:** 10.1155/2016/8589270

**Published:** 2016-02-23

**Authors:** Mahmoud Aljurf, Hala Abalkhail, Amal Alseraihy, Said Y. Mohamed, Mouhab Ayas, Fahad Alsharif, Hazza Alzahrani, Abdullah Al-Jefri, Ghuzayel Aldawsari, Ali Al-Ahmari, Asim F. Belgaumi, Claudia Ulrike Walter, Hassan El-Solh, Walid Rasheed, Maher Albitar

**Affiliations:** ^1^Oncology Centre, King Faisal Specialist Hospital and Research Centre, Riyadh 11211, Saudi Arabia; ^2^Pathology and Laboratory Medicine, King Faisal Specialist Hospital and Research Centre, Riyadh 11211, Saudi Arabia; ^3^Pediatric Hematology/Oncology, King Faisal Specialist Hospital and Research Centre, Riyadh 11211, Saudi Arabia; ^4^Hematology/Oncology, NeoGenomics Laboratories, Irvine, CA 92618, USA

## Abstract

*Background*. We studied DNA chimerism in cell-free DNA (cfDNA) in patients treated with HSCT.* Methods*. Chimerism analysis was performed on CD3+ cells, polymorphonuclear (PMN) cells, and cfDNA using 16 small tandem repeat loci. The resulting labeled PCR-products were size-fractionated and quantified.* Results*. Analyzing samples from 191 patients treated with HSCT for nonneoplastic hematologic disorders demonstrated that the cfDNA chimerism is comparable to that seen in PMN cells. Analyzing leukemia patients (*N* = 126) showed that, of 84 patients with 100% donor DNA in PMN, 16 (19%) had evidence of clinical relapse and >10% recipient DNA in the plasma. Additional 16 patients of the 84 (19%) showed >10% recipient DNA in plasma, but without evidence of relapse. Eight patients had mixed chimerism in granulocytes, lymphocytes, and plasma, but three of these patients had >10% recipient DNA in plasma compared to PMN cells and these three patients had clinical evidence of relapse. The remaining 34 patients showed 100% donor DNA in both PMN and lymphocytes, but cfDNA showed various levels of chimerism. Of these patients 14 (41%) showed laboratory or clinical evidence of relapse and all had >10% recipient DNA in cfDNA.* Conclusion*. Monitoring patients after HSCT using cfDNA might be more reliable than cellular DNA in predicting early relapse.

## 1. Introduction

Allogeneic hematopoietic stem cell transplantation (HSCT) is a potential curative treatment in patients with hematologic malignancies, many nonneoplastic hematologic disorders, and congenital immunodeficiency [1–3]. After several weeks of growth in the bone marrow, expansion of HSC and their progeny is sufficient to normalize the blood cell counts and reinitiate the immune system. Long-term reconstitution of donor cells predicts disease-free survival; however the detection of advancing host cells represents relapse, graft rejection, or failure [4–6]. Thus, in clinical transplantation settings monitoring donor cells engraftment and subsequent establishment of donor hematopoiesis is an important aspect in the evaluation of clinical outcome.

Chimerism analysis distinguishing donor from recipient in hematopoietic cell subsets (myeloid and lymphoid cells) after allogeneic HSCT is routine in the follow-up of patients. Chimerism analysis early after transplantation reflects engraftment kinetics, whereas analysis after engraftment assists the interpretation of clinical events such as graft-versus-host disease, secondary graft rejection, minimal residual disease, and disease relapse [7–10].

The basic principle in the detection of chimerism is the utilization of polymerase chain reaction (PCR) to analyze polymorphic genomic markers such as microsatellites/short tandem repeats (STR). STR are highly polymorphic, simple repetitive noncoding DNA that varies in number among different individuals. This method is well tested and has a detection limit of 1% to 5% [11–13]. Southern blot-based techniques (e.g., restriction-length fragment polymorphisms) are less sensitive and are not practical as compared with PCR-based techniques. Some testing is based on gender-mismatch. Transplantation has been used and this method shows fairly high sensitivity but this approach is limiting due to its dependence on gender.

The analysis of genomic DNA and free plasma DNA after organ transplantation has received significant attention for analysis of organ transplant tolerance and search for a noninvasive method to detect graft rejection [14, 15]. In solid organs, the release of donor cells and predominantly cell-free DNA into recipient circulation may be observed during episodes of graft rejection as a consequence of cell apoptosis during graft injury [16]. However, no work has been reported to address the utilization of cell-free circulating DNA after HSCT.

In allogeneic HSCT, the presence of 100% donor granulocytes and lymphocytes is an indication of a successful expansion of newly transplanted HSC and their progeny, which is sufficient to normalize the blood cell counts. When a relapse is taking place, we hypothesize that initially leukemic cells will grow and start taking over the hematopoietic cells causing reverse in the 100% donor engraftment. Since leukemic cells have higher turnover than normal cells, we hypothesize that their DNA can be detected in the plasma as free circulating DNA prior to being detected when lymphocytes or granulocytes are analyzed. In addition, usually the donor cells attempt to kill the leukemic cells in the well-documented graft-versus-leukemia (GVL) phenomenon and this may lead to the presence of free circulating pretransplant DNA in the plasma.

## 2. Methods

The study cohort included patients who had undergone allogeneic HSCT with conditioning regimen at King Faisal Specialist Hospital & Research Centre (KFSH & RC) and had routine post-HSCT chimerism testing of both granulocytes and lymphocyte in the peripheral blood. Stem cell grafts were from matched HLA siblings and rarely from matched unrelated donors or cord blood units. Peripheral blood samples were submitted for routine testing at different time points after transplantation. Lineage specific cells were sorted according to CD33 and CD3 expression. Chimerism analysis was performed to determine the relative ratio of donor DNA in comparison to recipient DNA in myeloid, lymphoid, and plasma samples.

### 2.1. Lineage Specific Separation

Prior to chimerism testing, myeloid and lymphoid cells were sorted using EasySep*™* (STEMCELL Technologies, Inc., Vancouver, Canada), positive selection human whole blood myeloid selection kit, and human whole blood CD3 positive selection kit, respectively. Myeloid and lymphoid cells were enriched separately by using immune-magnetic, column-free positive selection method following the manufacturer's instructions. Briefly, red blood cells were lysed and lymphoid cells were positively selected and then retained using magnetic nanoparticles. The magnetically labeled cells were washed and resuspended with phosphate buffered saline.

### 2.2. Nucleic Acid Extraction

DNA was extracted from separated cells using MagNA Pure LC system (Roche, Indianapolis, IN) automated system. Total nucleic acid was extracted from plasma samples using NucliSENS® easyMAG® automated system (bioMérieux) following the manufacturer's instructions.

### 2.3. Multiplex PCR

Myeloid, lymphoid, and plasma DNA was analyzed by multiplex fluorescence-based STR-PCR by using a combination of 16 microsatellite primers using AmpFLSTER® profiler kit (Applied Biosystems) following the manufacturer's instructions. The kit includes 15 labeled primers as well as primer for the amelogenin locus located on both X and Y chromosomes. The subsequent fluorescence labeling allows the detection of size-fractionated products on an automated 3130 Genetic Analyzer by using Performance Optimized Polymer 4 (Applied Biosystems). All DNA extraction and pre-PCR preparation were performed in restricted one-direction pre-PCR area. Positive, negative, reagent, and sample controls were run with every procedure. Assays were rejected and repeated if a discrepancy is observed between different tubes. Pretransplant samples were never processed at the same time when posttransplant samples were processed.

### 2.4. Chimerism Analysis

Extracted data was analyzed by GeneScan software version 3.12 (Applied Biosystems). The results of chimerism were reported as donor cells percentages in neutrophil granulocyte and lymphocyte lineages. The donor percentage peak area was calculated as % donor: (donor peaks/donor + recipient peaks) × 100. The calculation is made only from informative alleles that distinguish donor from recipient.

### 2.5. Statistical Analysis

The correlations were calculated using Spearman rank correlation coefficients. The Wilcoxon rank sum test or Kruskal-Wallis test was used to compare among categorical variables. *P* values less than 0.05 were considered statistically significant.

## 3. Results

### 3.1. Circulating Cell-Free DNA Is Generated Mainly from Polymorphonuclear Cells

We analyzed chimerism in plasma, lymphocytes, and granulocytes from 191 patients transplanted for causes other than neoplastic diseases and 126 patients transplanted for neoplastic diseases. The nonneoplastic diseases included thalassemia, immune deficiencies, sickle cell anemia, and other congenital abnormalities. The neoplastic diseases included acute lymphoblastic leukemia (ALL), acute myeloid leukemia (AML), chronic myeloid leukemia (CML), myeloproliferative diseases, and various types of lymphoma. The neoplastic patients included 97 (51%) adults and 94 (49%) pediatric patients.

**Figure 1:**
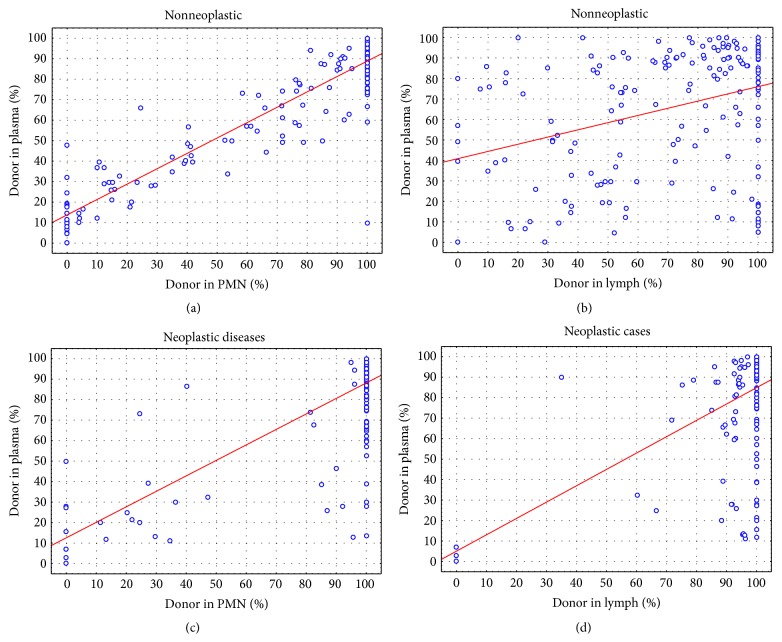
Comparing percent of donor DNA in plasma cfDNA with polymorphonuclear (PMN) and lymphocytes. (a) cfDNA versus PMN DNA in patients with nonneoplastic diseases; (b) cfDNA versus Lymphocytes DNA in patients with nonneoplastic diseases; (c) cfDNA versus PMN in patients with hematologic neoplasms; (d) cfDNA versus lymphocyte DNA in patients with hematologic neoplasms.

We first compared plasma with polymorphonuclear cells (PMN) levels of donor DNA in nonneoplastic samples. As seen in Figure 1(a), there was significant correlation (Spearman, *P* < 0.0001) in relative donor DNA between the two sample types. However, the relative donor DNA was slightly at higher level in plasma as compared with PMN cells. The correlation was significantly less between the lymphocytes and cell-free plasma levels of donor DNA in the nonneoplastic samples (Figure 1(b)). More importantly, donor DNA was overall relatively significantly higher in the cell-free plasma DNA as compared with cellular DNA in both lymphocytes and PMN.

In patients transplanted for neoplastic causes, the correlation was less between the plasma and PMN. However, the donor DNA (Figure 1(c)) was at higher level in the plasma as compared with PMN. There was significantly better correlation between plasma and PMN in relative donor DNA level as compared with lymphocytes (Figure 1(d)).

This data suggests that most of the DNA in the plasma is produced by the turnover of the PMN cells. In nonneoplastic disease, the correlation between plasma and PMN is very high because the cells are similar and their turnover is similar. In neoplastic diseases, the DNA in the plasma could be due to simple chimerism and neutrophils are pouring their DNA from both donor and recipient at equal level in some patients but could also be due to neoplastic cells that are contributing a higher level of recipient DNA due to the high turnover of the tumor cells.

### 3.2. Plasma Is More Sensitive Than Cells in Detecting Relapse of Leukemia

We analyzed patients transplanted as a treatment for leukemia (*N* = 84) who had 100% donor DNA in their PMN. Of these patients 50 (59.5%) had various levels of recipient DNA in the plasma (*P* < 0.0001 Sign test) and 28 (33%) had recipient DNA levels >10% (*P* < 0.0001 Sign test). Of the 84 patients with 100% donor DNA in PMN, 16 (19%) patients had clinical evidence of relapse. All patients with relapse had >10% recipient DNA in the plasma reflecting the relapsing leukemic cells. However, additional 16 patients showed more recipient DNA (>10%) in plasma, but without evidence of relapse. In addition, 8 patients had mixed chimerism in granulocytes and lymphocytes as well as plasma, but 3 of these patients had >10% recipient DNA in plasma compared to granulocytes and these 3 patients had evidence of relapse.

When we considered all patients who had 100% donor DNA in both lymphocytes and PMN, but with donor plasma DNA levels between 10% and 99% (#34) (Table 1), 14 out of 34 (41%) patients had evidence of disease either clinically or by cytogenetic or molecular means (Table 1). One patient died and was considered clinically in remission, but no autopsy was performed. Two of the relapses were extramedullary: one is mediastinal and the other is testicular.

**Figure 2:**
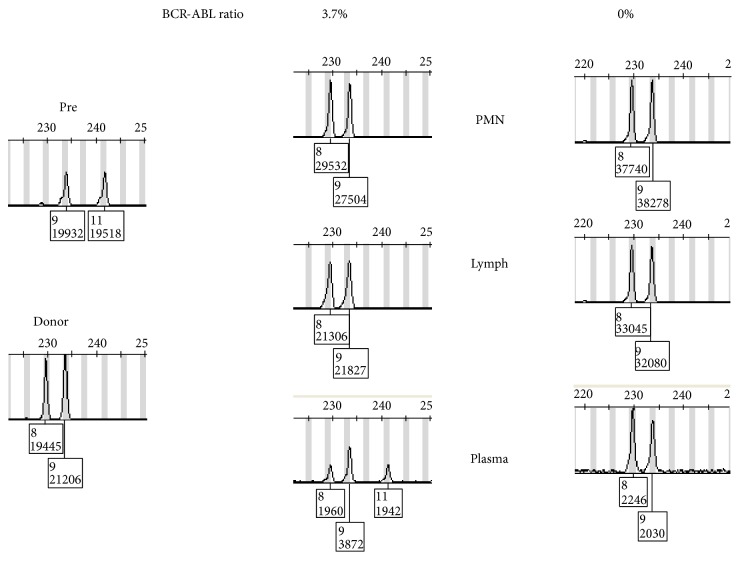
Chimerism pattern in a patient with CML treated with HSCT. Pretransplant and donor patterns are shown in the left panel. The middle and right panels show the patterns in PMN, lymphocytes, and plasma at two different points. The BCR-ABL fusion level as detected by RT/PCR is shown on top. The middle sample shows mixed chimerism in plasma only as well as residual BCR-ABL1 fusion RNA. The right panel shows the disappearance of the recipient DNA and fusion BCR-ABL1.

An example of chimerism pattern in a CML patient is shown in Figure 2 with positive low-level BCR-ABL: ABL transcript ratio. Meanwhile chimerism study showed 100% donor DNA in lymphocytes and polymorphonuclear cells, but plasma showed mixed chimerism. Later testing showed disappearance of the recipient DNA coinciding with disappearance of BCR-ABL fusion transcript.

## 4. Discussion

The standard method of chimerism analysis after allogeneic HSCT relies on DNA testing by PCR methods of selected cell subsets (myeloid and lymphoid). Performing chimerism analysis on other cell subsets, such as plasma cells in myeloma patients, after HSCT has also been reported [14]. However, there are no reports on cfDNA-based chimerism testing in patients after HSCT. In this single centre series, we report our observations for chimerism analysis using cfDNA and compare this to the standard cell subset testing method.

Chimerism analysis after allogeneic HSCT is reported to allow early detection of patients at high risk of clinical relapse [10, 17, 18]. Early detection of minimal residual disease status after HSCT has prognostic implications as well as possible early therapeutic interventions such as donor lymphocyte infusion (DLI).

Our data suggests that most of the cfDNA in the plasma is generated from the turnover of polymorphonuclear cells. Furthermore, due to the high turnover of leukemic cells and pouring this DNA into plasma, the plasma may show evidence of relapse at an early stage. Patients with 100% donor DNA in both lymphocytes and PMN but less than 90% donor DNA in plasma should be closely watched for the potential of relapse. This 10% of recipient DNA cut-off point is an arbitrary number that is relevant in this set of patients. Considering the sensitivity of the assay is in the range of the 5% and using 10% to establish significant difference is a logical approach, but further studies with large number of patients and more detailed longitudinal data are needed to precisely determine the exact cut-off point as well as the sensitivity and specificity of cfDNA in predicting relapse. Most likely higher levels of recipient DNA correlate with more imminent relapse than lower level. Furthermore there might be a difference between patients dependent on the type of disease. This is particularly relevant when there are no specific, easy to follow biomarkers or molecular abnormalities in the leukemic cells. Combining plasma-based detection of chimerism with the plasma-based detection of specific mutations which was detected in the leukemic cells might also add another level to improve early detection of relapse. Further studies are needed to dissect the clinical relevance and clinical application of such approach in managing patients after HSCT.

## Figures and Tables

**Table 1:** Patients with 100% donor chimerism in lymphocytes and PMN with mixed (donor and recipient) plasma chimerism.

Patient	PMN	Lymph	Plasma donor ratio	Diagnosis	Status
1	100	100	30	AML	Death in remission
2	100	100	38.8	CML	BCR-ABL: 0.56
3	100	100	52.5	CML	BCR-ABL: 3.7
4	100	100	57	AML	EM disease
5	100	100	60	CML	BCR-ABL: 0.003
6	100	100	64.5	ALL	Relapse
7	100	100	66.9	AML	Relapse
8	100	100	68.4	ALL	FISH 3% monosomy 17
9	100	100	69.4	ALL	Relapse
10	100	100	74.4	CML	CR
11	100	100	75	CML	BCR-ABL: 0.03
12	100	100	76	AML	CR
13	100	100	76.2	ALL	10% by FISH t(1; 19)
14	100	100	76.5	ALL	CR
15	100	100	78.3	CML	BCR-ABL: 3.57 one month earlier
16	100	100	79.9	ALL	CR
17	100	100	80	AML	CR
18	100	100	80	AML	CR
19	100	100	80	ALL	CR
20	100	100	81.7	ALL	CR
21	100	100	82	AML	CR
22	100	100	82.4	AML	CR
23	100	100	84.3	AML	Relapse
24	100	100	84.6	AML	CR
25	100	100	84.7	AML	CR
26	100	100	88.1	ALL	CR
27	100	100	88.4	ALL	EM disease
28	100	100	89.3	AML	CR
29	100	100	89.4	AML	CR
30	100	100	90	CML	CR
31	100	100	90	AML	Relapse
32	100	100	90	ALL	CR
33	100	100	90	ALL	CR
34	100	100	90	AML	CR

PMN: polymorphonuclear cells; EM: extramedullary.
